# BMSC-derived exosomes promote tendon-bone healing after anterior cruciate ligament reconstruction by regulating M1/M2 macrophage polarization in rats

**DOI:** 10.1186/s13287-022-02975-0

**Published:** 2022-07-15

**Authors:** Zhenyu Li, Qingxian Li, Kai Tong, Jiayong Zhu, Hui Wang, Biao Chen, Liaobin Chen

**Affiliations:** 1 Division of Joint Surgery and Sports Medicine, Department of Orthopedic Surgery, Zhongnan Hospital of Wuhan University, Wuhan, China; 2 Department of Pharmacology, Wuhan University Taikang Medical School (School of Basic Medical Sciences), Wuhan University, Wuhan, 430071 China; 3 Hubei Provincial Key Laboratory of Developmentally Originated Disease, Wuhan, 430071 China

**Keywords:** Bone marrow stromal cell, Exosome, Macrophage polarization, Tendon-bone healing, miRNA

## Abstract

**Background:**

Recent studies have shown that bone marrow stromal cell-derived exosomes (BMSC-Exos) can be used for tissue repair. However, whether the BMSC-Exos can promote tendon-bone healing after anterior cruciate ligament reconstruction (ACLR) is still unclear. In this study, we observed in vivo and in vitro the effect of rat BMSC-Exos on tendon-bone healing after ACLR and its possible mechanism.

**Methods:**

Highly expressed miRNAs in rat BMSC-Exos were selected by bioinformatics and verified *in vitro*. The effect of overexpressed miRNA in BMSC-Exos on M2 macrophage polarization was observed. A rat model of ACLR was established. The experimental components were divided into three groups: the control group, the BMSC-Exos group, and the BMSC-Exos with miR-23a-3p overexpression (BMSC-Exos mimic) group. Biomechanical tests, micro-CT, and histological staining were performed for analysis.

**Results:**

Bioinformatics analysis showed that miR-23a-3p was highly expressed in rat BMSC-Exos and could target interferon regulatory factor 1 (IRF1, a crucial regulator in M1 macrophage polarization). In vitro, compared with the control group or the BMSC-Exos group, the BMSC-Exos mimic more significantly promoted the polarization of macrophages from M1 to M2. In vivo, at 2 weeks, the number of M2 macrophages in the early local stage of ACLR was significantly increased in the BMSC-Exos mimic group; at 4 and 8 weeks, compared with the control group or the BMSC-Exos group, the bone tunnels of the tibia and femur sides of the rats in the BMSC-Exos mimic group were significantly smaller, the interface between the graft and the bone was narrowed, the bone volume/total volume ratio (BV/TV) increased, the collagen type II alpha 1 level increased, and the mechanical strength increased.

**Conclusions:**

BMSC-Exos promoted M1 macrophage to M2 macrophage polarization via miR-23a-3p, reduced the early inflammatory reaction at the tendon-bone interface, and promoted early healing after ACLR.

**Supplementary Information:**

The online version contains supplementary material available at 10.1186/s13287-022-02975-0.

## Background

The anterior cruciate ligament (ACL), located inside the knee joint, plays a vital role in limiting anterior tibia translation and controlling axial knee rotation and varus movement. Recent evidence suggests that ACL injury is one of the most frequent knee injuries [1], with an incidence of 68.6 per 100,000 person-years [2]. An ACL injury can result in recurring knee instability, meniscus tears, and even cartilage injury, increasing the risk of osteoarthritis and disability [3]. Hence, early treatment of ACL injury is essential for maintaining joint health. Currently, ACL reconstruction (ACLR) is regarded as the best treatment after an ACL injury. However, a long time is required to return to sports for patients who undergo ACLR because of the slow natural healing of tendon-bone [4], and even poor healing after ACLR may lead to reinjury of the ACL, which dramatically increases the pain of patients [5]. Therefore, ensuring rapid, effective, and stable tendon-bone healing after ACLR and restoring knee joint function as early as possible is a critical issue that urgently needs to be resolved.

Successful tendon-bone healing after ACLR requires mechanical and biological enhancement of the femur-graft-tibia complex to maintain the stability of the knee joint. Although minimally invasive surgery aims to reduce surgical trauma to the body, the local inflammatory response of the bone tunnel induced by surgery inhibits the tendon-bone healing process after ACLR [6]. In the progression of inflammation, the crucial role of macrophages cannot be ignored. Macrophages are classified into M1 macrophages and M2 macrophages. After ACLR, monocyte-derived macrophages can polarize into M1 macrophages, which are characterized by their proinflammatory function and can phagocytose apoptotic cells and remove cell debris [7]. However, M1 macrophages are gradually replaced by M2 macrophages over time, and M2 macrophages display anti-inflammatory functions. Therefore, accelerating the promotion of M1 to M2 polarization of macrophages can speed up tissue repair [8]. If macrophages are not converted to M2 in time, it may lead to a prolonged local inflammation period in the wound and eventually lead to slower healing [9]. Therefore, modulation of the polarization state of macrophages may be a valuable strategy to promote early healing in ACLR.

Bone marrow-derived mesenchymal stem cells (BMSCs), a type of adult stem cell derived from the mesoderm, have immunomodulatory properties and regulate various immune cells involved in the immune response [10]. In recent years, there has been increasing evidence that BMSCs therapy is effective in treating joint diseases, such as cartilage lesions and osteoarthritis [11]. The regulatory effect of BMSCs on inflammation is accomplished by delivering specific cargoes to recipient cells via the paracrine ability of exosomes [12]. Exosomes are nanosized (30-150 nm) bilayer membrane extracellular vesicles wrapped with small RNAs and proteins, which are recognized as important mediators in intercellular genetic exchange and communication [13, 14]. It has reported that BMSC-derived exosomes (BMSC-Exos) reduce dextran sodium sulfate-induced inflammatory bowel disease in mice via the polarization of M2 macrophages [15]. Another study elucidated that miR-182 in BMSC-Exos attenuated myocardial ischemia–reperfusion injury by polarizing macrophages toward M2 macrophages [16]. Recently, researchers found that stem cell-derived exosomes improve the healing speed of tendon repair [17, 18] and ameliorate the chronic rotator cuff tendinopathy [18]. However, there are differences between ACL and rotator cuff or tendon in tissue function and loading characteristics, whether BMSC-Exos can regulate the polarization of macrophages at the ACLR tendon-bone interface and the local inflammatory environment to promote the early tendon-bone healing of ACLR has not been reported.

In this study, we aimed to investigate the effect of BMSC-Exos on M2 polarization and reveal its mechanism in vitro and in vivo. Our findings may provide a new strategy to accelerate patients' recovery from ACLR in future.

## Methods

### Chemicals and reagents

Macrophage colony stimulating factor (M-CSF) and interferon-gamma (IFN-γ) were purchased from MedChemExpress (Shanghai, China). Lipopolysaccharides (LPS) was purchased from Sigma-Aldrich (St. Louis, MO, USA). α-MEM, fetal bovine serum (FBS), penicillin-streptomycin, and Lipofectamine 3000 were purchased from Gibco (Waltham, MA, USA). Osteogenic differentiation medium, adipogenic differentiation medium, and chondrogenic differentiation medium were purchased from Procell Life Science & Technology Co, Ltd (Procell, Wuhan, China).1,1′-dioctadecyl-3,3,3′,3′-tetramethylindocarbocyanine perchlorate (DiI) and 4′,6-diamidino-2-phenylindole (DAPI) were purchased from Beyotime (Shanghai, China). TRIzol reagent was purchased from Invitrogen (Carlsbad, CA). miR-23a-3p mimic, miR-23a-3p inhibitor and their respective negative controls were purchased from GeneChem (Shanghai, China). MiScript reverse transcription kit and miScript SYBR Green PCR Kit were purchased from QIAGEN (Hilden, Germany). cDNA Reverse Transcription Kit and SYBR Green PCR kit were purchased from Vazyme (Nanjing, China). Antibodies against CD9 (A1703), CD63 (A5271), tumor susceptibility gene 101 (TSG101) (A5789), Calnexin (A15631), inducible nitric oxide synthase (iNOS) (A0312), arginase 1 (Arg1) (A1847), interferon regulatory factor 1 (IRF1) (A7692), P-P65 (AP0123), P65 (A2547), CD68 (A6554), glyceraldehyde-3-phosphate dehydrogenase (GAPDH) (AC002), HRP goat anti-rabbit IgG (AS014), and Cy3 goat anti-rabbit IgG (AS007) were purchased from Abclonal (Wuhan, China). Antibodies against CD163 (16646-1-AP) and collagen type II alpha 1(COL2α1) (28459-1-AP) were purchased from Proteintech (Wuhan, China).

### Experimental animals

All Wistar rats were purchased from Hubei Provincial Centers for Disease Control and Prevention (Wuhan, China) (No. 42000600040610). Our research was approved by the Medical Ethics Committee for Experimental Animals of Zhongnan Hospital of Wuhan University (Wuhan, China) (Approval No. ZN2021061) according to the Guide for the Care and Use of Laboratory Animals. All animals were kept under standard housing conditions (12-h light/dark cycle, humidity 40–60%, and temperature 22 °C) with free access to food and water. After adaptive feeding for one week, the animals were used for the subsequent experiments.

### Cell isolation and culture

BMSCs were isolated from 4-week-old Wistar rats and cultured as described in our previous study [19]. Briefly, we isolated the tibia and femur of rats and then flushed the bone marrow cavity with a syringe. The cells were lysed in red blood cell lysate and cultured in 10% FBS, 100 U/ml penicillin and 100 µg/ml streptomycin at 37 °C and 5% CO_2_. The medium was replaced every two days. BMSCs were used in subsequent experiments within 5 generations. Bone marrow-derived macrophages (BMDMs) were also flushed through the bone marrow cavity of the rat femur and tibia. Briefly, after the cells were flushed out of the bone marrow cavity and red blood cell lysate, they were cultured in a T25 culture flask for 24 h to isolate adherent cells. Then, 50 ng/ml M-CSF was added for 7 days to induce mononuclear macrophages. The medium was changed every two days. For M1 polarization, we stimulated BMDMs with 100 ng/ml LPS and 40 ng/ml IFN-γ for 24 h.

### The trilineage differentiation ability of BMSCs

Trilineage differentiation of BMSCs was performed in a similar way to that in previous studies [20–22]. The third cell passage was used for differentiation.

Specifically, for adipogenic differentiation: when the BMSCs confluence was close to 100%, the adipogenic differentiation medium (Procell, Wuhan, China) was replaced, and the medium was changed every three days. On day 21, cells were fixed with 4% paraformaldehyde and then stained with Oil Red O for 30 min to detect adipogenic differentiation.

For osteogenic differentiation: when the BMSCs confluence reached 70%, the osteogenic differentiation medium (Procell, Wuhan, China) was changed, and the medium was changed every three days. On day 21, after cells were fixed with 4% paraformaldehyde, osteogenic differentiation was detected by Alizarin Red S staining for 30 min.

For the 3D pellet culture of chondrogenic differentiation: when BMSCs confluence reached 80%, cells were digested with trypsin, centrifuged to make the pellet of BMSCs, and the medium was replaced with chondrogenic differentiation medium (Procell, Wuhan, China). The culture medium was changed every three days. On day 21, after fixing the cells with 4% paraformaldehyde, the pellet was embedded in paraffin and sectioned. The chondrogenic differentiation was detected by Alcian blue staining for 30 min.

### Isolation and characterization of exosomes

To extract exosomes, we changed the medium to serum-free medium when BMSCs were approximately 70% confluent. The exosomes were collected by ultracentrifugation. Briefly, after culturing with FBS-free medium for 48 h, we collected the culture supernatant, centrifuged them at 200 ×g at 4 °C for 10 min to remove dead cells, centrifuged them at 2000 ×g for 20 min to remove cell debris, and then centrifuged the culture supernatant at 10,000 ×g for 1 h to remove broken cells and organelles. The obtained supernatant was passed through a 0.22 μm filter to remove larger particles. Then, the exosomes were obtained by centrifugation at 110,000 ×g at 4 °C for 70 min. After centrifugation, the exosomes were washed once with phosphate-buffered saline (PBS) and then centrifuged at 110,000 ×g at 4 °C for 70 min. Finally, the obtained exosomes were resuspended in PBS and used for subsequent analysis.

The characterization of exosomes was observed by transmission electron microscopy (TEM). We used nanoparticle tracking analysis (NTA) to analyze the particle size and distribution. Exosome-specific biomarkers CD9, CD63, and TSG101 were detected by Western blot. For the quantification of exosomes, we used the bicinchoninic acid (BCA) method for total protein determination.

### Exosome labeling and cellular uptake

To perform exosomal uptake experiments, BMSC-Exos were labeled with DiI and then washed with PBS at 110,000 ×g for 70 min at 4 °C. Then, BMSC-Exos stained with DiI were added to macrophages for 6 h at a concentration of 50 µg/ml. After 6 h, the cells were washed with PBS, and the nuclei were stained with DAPI. To exclude nonspecific staining, when collecting exosomes for the last time, we collected the same volume of supernatant and performed DiI staining as a sham control. Finally, images were taken using a confocal microscope (Smartproof 5, Carl Zeiss, Germany).

### Transfection and luciferase report assay

For the transfection experiment, BMSCs were transfected with 50 nmol/l miR-23a-3p mimic, 200 nmol/l miR-23a-3p inhibitor or their respective negative controls with Lipofectamine 3000 according to the manufacturer's method. After transfection, the BMSCs were cultured for 48 h. Then, we collected exosomes of BMSC-Exos transfected with negative control (BMSC-Exos NC), BMSC-Exos with miR-23a-3p overexpression (BMSC-Exos mimic), and BMSC-Exos with miR-23a-3p inhibitor (BMSC-Exos inhibitor) respectively.

For the luciferase reporter assay, firefly and Renilla luciferase reporter plasmids were obtained from GeneChem (Shanghai, China). 293T cells were co-transfected with wild-type or mutant IRF1 plasmids and miR-23a-3p mimic/negative controls for 8 h using Lipofectamine 3000. After 24 h, the luciferase activity of cells was measured using the Promega Dual-Luciferase system. The relative luciferase activity was calculated as firefly to Renilla luciferase luminescence.

### Cellular immunofluorescence (IF)

To determine the protein expression in cells, we used cellular IF. Specifically, after giving different treatments, we fixed the cells with 4% paraformaldehyde for 15 min, then permeabilized the cellular membrane with 0.3% Triton X-100 for 15 min, and washed with PBS 3 times. Then, the cells were blocked with 3% BSA for 1 h at room temperature. Incubation with primary antibody was performed overnight at 4 °C. On the next day, after washed with PBS 3 times, the cells were incubated with secondary antibody and DAPI at room temperature. The final image was taken with a confocal microscope (Smartproof 5, Carl Zeiss, Germany). The staining intensity was determined by measuring the integrated optical density (IOD) in 5 different fields for each sample. The fluorescence IOD value was obtained by calculating the total reaction intensity of all selected objects (target proteins) in a specific field of vision by Image J software.

### Reverse transcription-PCR

We used TRIzol to extract total RNA from exosomes and cells according to the manufacturer's procedures. For the detection of miRNA, we used a MiScript reverse transcription kit for reverse transcription and used miScript SYBR Green PCR Kit to identify the miRNA content according to the following volume and procedures: reaction mixture (20 μl), consisting of 10 μl 2x QuantiTect SYBR Green PCR Master Mix, 2 μl of 10x miScript Universal Primer, 2 μl of primer of miRNA, 4 μl of RNase-free H_2_O and 2 μl of cDNA templates; 15 min at 95 °C; 35 cycles of 15 s at 94 °C, 30 s at 55 °C, and 30 s at 70 °C. Primers for U6 small nuclear RNA-2 (RNU6-2) was purchased from QIAGEN and was used as an internal control. For the expression of mRNA, we used cDNA Reverse Transcription Kit for reverse transcription, and used a SYBR Green PCR kit on an ABI Step One Plus cycler (Applied Biosystems, Foster City, CA, USA) to identify the mRNA content according to the following volume and procedures: reaction mixture (20 μl), consisted of 10 μl of 2×SYBR quantitative polymerase chain reaction (qPCR) Mix, 1 μl of forward primer, 1 μl of reverse primer, 6 μl of RNase-free H_2_O and 2 μl of cDNA templates; 10 min at 95 °C; 35 cycles of 15 s at 95 °C, 20 s at 60 °C and 15 s at 72 °C. The relative expression level of GAPDH was used as an internal reference and expressed by the double delta CT method. The primers used in this experiment are all shown in Table 1.

**Table 1:** Oligonucleotide primers in real-time quantitative PCR.

Gene	Forward primer	Reverse primer
iNOS	TCCTCAGGCTTGGGTCTTGT	ATCCTGTGTTGTTGGGCTGG
IL-6	AGGATACCACCCACAACAGACC	TTGCCATTGCACAACTCTTTTC
TNF-α	AACAAGGAGGAGAAGTTCCCAAA	CTCCTCCGCTTGGTGGTTT
IL-1β	GCTTCCTTGTGCAAGTGTCT	TCTGGACAGCCCAAGTCAAG
Arg1	CAAGCCAAAGCCCATAGAGATT	CATTGGCTTTTCCCACAGACC
IL-10	CGACGCTGTCATCGATTTCTC	CAGTAGATGCCGGGTGGTTC
CD163	GACAGACCCAACGGCTTACA	GGTCACAAAACTTCAACCGGA
TGF-β	CTGCTGACCCCCACTGATAC	AGCCCTGTATTCCGTCTCCT
GAPDH	AGTGCCAGCCTCGTCTCATA	GGTAACCAGGCGTCCGATAC
miR-23a-3p	AGATCACATTGCCAGGGAT	miScript Universal Primer^a^
miR-130a-3p	CAGCAGTGCAATGTTAAAAGG	miScript Universal Primer^a^

iNOS, inducible nitric oxide synthase; IL-6, Interleukin 6; TNF-α, tumor necrosis factor α; IL-1β, Interleukin 1β; Arg1, arginase 1; IL-10, Interleukin 10; TGF-β, transforming growth factor β; GAPDH, glyceraldehyde-3-phosphate dehydrogenase.

^a^The miScript Universal Primer in the miScript SYBR Green PCR Kit was used as the reverse primer.

### Western blot analysis

RIPA was used to extract the total protein of the exosomes and cells. Then, we used SDS-PAGE gel for protein semi-quantitative analysis and used PVDF membrane for transfer. Incubating the strips at 4 °C overnight. The next day, after incubating the corresponding HRP-labeled secondary antibody, the ECL luminescence kit was used to detect the expression level on a gel imaging system (Tanon Science and Technology Co. Ltd, Shanghai, China). GAPDH was used as an internal reference, and ImageJ was used for semi-quantitative analysis.

### ACLR surgery model building

Ninety 8-week-old male Wistar rats underwent unilateral ACL resection followed by isometric ACLR. Briefly, the rats were anesthetized with pentobarbitone sodium (40 mg/kg intraperitoneally). After skin disinfection and shaving, the peroneus longus tendon was harvested from the outside of the right ankle joint. Then, 4-0/T non-absorbable was used to suture both ends for ACLR. Once the autogenous tendon is prepared, we use medial patellar arthrotomy to open the right knee joint and remove the autogenous ACT. Then, we used a 0.9 mm drill to create bone tunnels on the femoral and tibial sides. The graft was fixed on the outside of the bone tunnel with surgical sutures, and the sutures were tied to the periosteum with a 5 N load [23]. After the operation, the incision was sutured, and the rats were allowed to move and eat freely.

All rats were randomly divided into 3 groups: the control group (*n* = 30), the BMSC-Exos group (*n* = 30), and the BMSC-Exos mimic group (*n* = 30). For the control group, the rats received 50 µl PBS injection into the joint cavity after the operation, 3 days after the operation and 7 days after the operation. For the BMSC-Exos group and BMSC-Exos mimic, injections of the same volume of BMSC-Exos (10^11^ particles/ml) and BMSC-Exos mimic (10^11^ particles/ml) were performed after the operation, 3 days after the operation and 7 days after the operation, respectively. Before each injection, we flexed the knee joint of the rat by 90° to obtain more joint space and performed it at the middle and midline of the patellar tendon to avoid damage to the ligaments in the joint.

### Biomechanical testing

To detect the healing of tendon to bone after ACLR, we performed biomechanical tests at 4 and 8 weeks after the operation using a mechanical tester (ElectroForce 3220, Bose, ID, USA). After separating the tibia-graft-femoral complex, we removed the muscles around the joint and the remaining ligaments, leaving only the reconstructed ACL. Then, the tissue was fixed firmly on the mechanical tester. Samples were preloaded statically by 0.5 N for 1 min. Then, the ultimate failure load was documented at an elongation rate of 3 mm/min. The load was recorded when the sample was broken or pulled out and recorded as the maximum tensile force (N). Then, the stiffness (N/mm) of the sample is expressed by the slope of the linear part on the load-deformation curve. Throughout the test, all samples were kept moist with normal saline.

### Micro-CT analysis

The micro-CT scan was performed perpendicular to the long axis of the sample through the SkyScan 1276 micro-CT device (Bruker micro-CT, Kontich, Belgium). The parameters of micro-CT are as follows: resolution ratio, 20.3 μm; X-ray source voltage, 60 kV; X-ray source current, 200 μA. For the cross-sectional areas of the femoral and tibial sides of the sample, we used the 2 weeks control group as a control. For the bone volume/total volume ratio (BV/TV), we analyzed a cylinder-shaped region of interest with a diameter of 1.2 mm and height of 2 mm, covering the proximal end of the tibial tunnel or the distal end of the femoral tunnel. Subsequently, the sample was decalcified with 10% ethylenediaminetetraacetic acid (EDTA) solution.

### Histology staining

The tissues after micro-CT scanning were decalcified, embedded in paraffin, and cut to 4 μm sections. The sections were deparaffinized and hydrated, and HE was performed according to the manufacturer's methodology. Then the slices were viewed using PhenoChart software (PerkinElmer, Inc.). The mean interface width of a sample was calculated by averaging the width at every of the tunnel’s cross section, and three sections were measured per sample. A thinner tendon-bone interface indicates better integration [24–27].

Slices were stained with Safranin-O/Fast Green for analysis of the formation of chondrocytes and fibrocartilage at the tendon-bone interface according to the manufacturer’s instructions. The images were captured with a Nikon NIS Elements BR light microscope (Nikon, Tokyo, Japan).

### Immunohistochemistry (IHC) of tissue sections

For IHC, we cut the decalcified and embedded tissue into 4 μm sections. Then, we dewaxed the paraffin to water, and used citric acid antigen retrieval solution for antigen retrieval. After that, the sections were incubated in the dark at room temperature for 25 min to block endogenous peroxidase. After washing 3 times with PBS, 3% BSA was used to block for 30 min at room temperature. Then the sections were incubated with the primary antibody overnight at 4°C. The next day, the sections were incubated with the HRP-conjugated secondary antibody. Finally, a DAB kit was used for color development. The nucleus was stained with hematoxylin. Finally, the slices were dehydrated and mounted, and the images were captured with a Nikon NIS Elements BR light microscope (Nikon, Tokyo, Japan).

### IF staining of tissue sections

For IF staining, we deparaffinized the sections to water, and then performed antigen retrieval in citric acid antigen retrieval solution. After the antigen retrieval of the slices was completed, they were blocked with 3% BSA for 1 h and then incubated with the primary antibody at 4 °C overnight. On the second day, after incubating the fluorescent secondary antibody, the nucleus was stained with DAPI. The slides were observed and photographed by a Nikon fluorescence microscopy (Nikon, Tokyo, Japan).

### Statistical analysis

The GraphPad Prism 9.0 software (San Diego, CA) was used for all statistical analyses. All data were expressed as mean ± SD. n values represented the number of independent experiments or the number of animal samples used. To detect a statistical difference among groups regarding maximal failure load, the number of rats per group was required based on a power analysis with *β* = 0.8 and *α* = 0.05. All groups were compared with Student *t* test for two-group comparisons, or one-way ANOVA analysis of variance followed by Turkey’s post hoc test for multiple comparisons. *P* < 0.05 were considered statistically significant.

## Results

### The identification of rat BMSCs and BMSC-Exos

**Fig. 1:**
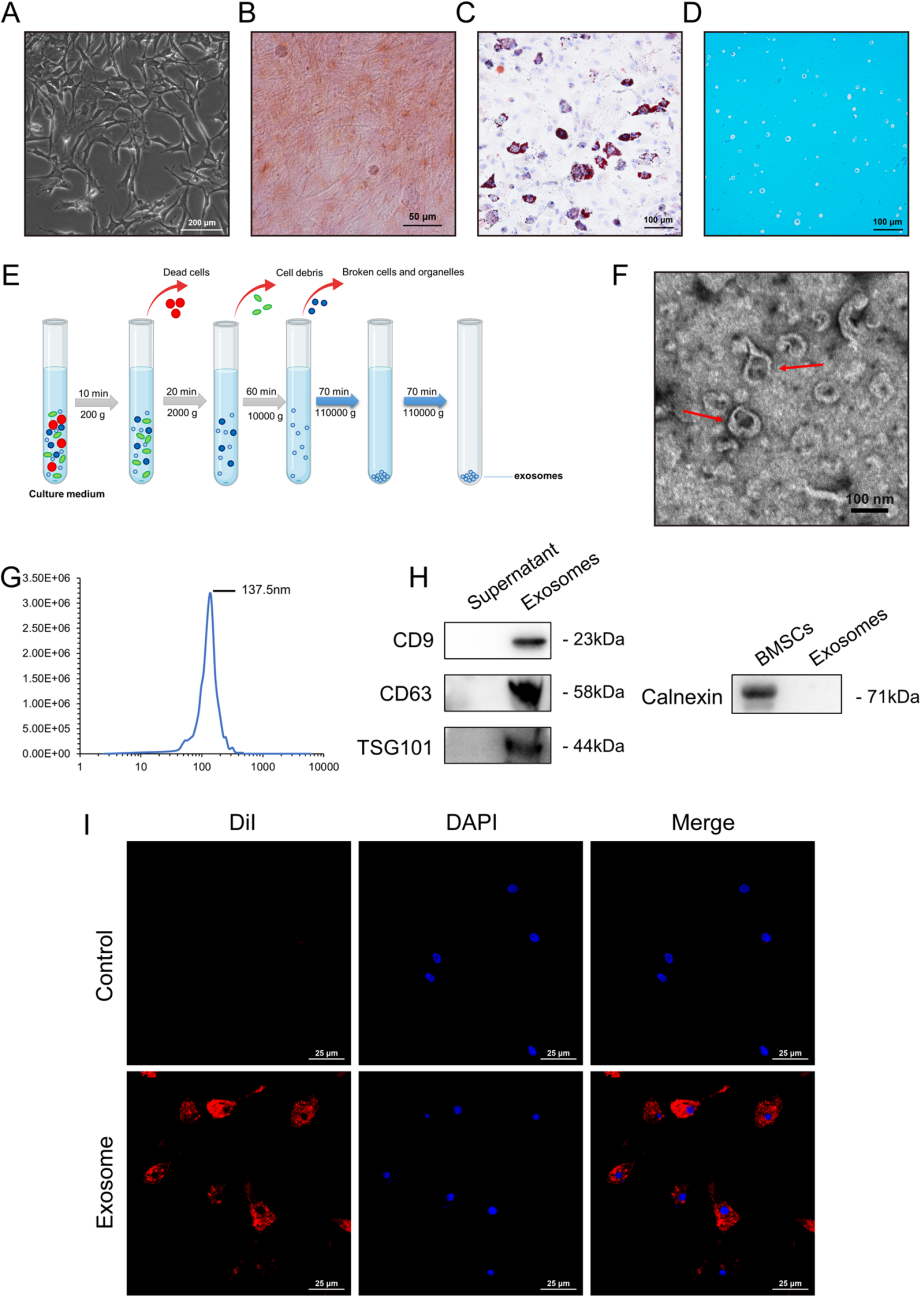
Morphology and characterization of BMSCs and BMSC-Exos. **A** Representative light microscopic images of BMSCs (Scale bar: 200 μm). The identification of osteogenic, adipogenic, and chondrogenic differentiation potential of BMSCs, and the representative microscopic image of Alizarin Red S staining (**B**) (Scale bar: 50 μm), Oil Red O staining (**C**) (Scale bar: 100 μm), and Alcian blue staining (**D**) (Scale bar: 100 μm). *n* = 3. **E** Schematic diagram of the isolation of the BMSC-Exos. **F** Transmission electron microscopy (TEM) image of BMSC-Exos. Scale bar: 100 nm. **G** NanoSight tracking analysis (NTA) for the diameters of BMSC-Exo. **H** Western blot analysis of CD9, CD63, TSG101, and Calnexin. *n* = 3. **I** Analysis of cellular exosome uptake by macrophages. *n* = 3. Scale bar: 25 μm.

First, we identified BMSCs extracted from the rat bone marrow cavity. The BMSCs exhibited a spindle-like morphology (Fig. 1A) under light microscopy. Alizarin Red S staining (Fig. 1B), Oil Red O staining (Fig. 1C), and Alcian blue staining (Fig. 1D) showed that the BMSCs could differentiate into osteoblasts, adipocytes, or chondrocytes. Next, to characterize the exosomes derived from BMSCs, they were isolated and purified from the culture supernatants of BMSCs by ultracentrifugation (Fig. 1E) and then comprehensively characterized via TEM, NTA, and Western blot. TEM data showed that these vesicles mostly had a typical spherical double-membrane structure with a diameter of approximately 100 nm (Fig. 1F). NTA demonstrated that the diameter size distribution of these vesicles showed a peak value of 137.5 nm (Fig. 1G). Then, Western blot results showed that the maker proteins of the exosome membrane, CD9, CD63, and TSG101, were expressed in exosomes compared to the supernatant (Fig. 1H). And the Calnexin (an endoplasmic reticulum protein) was not expressed in exosomes (Fig. 1H), indicating that the isolated exosomes were free of contamination with cellular components. Moreover, the cell membrane fluorescent probe DiI was used to label BMSC-Exos. The labeled BMSC-Exos were added to the culture medium of macrophages. After incubation for 6 h, DiI-labeled exosomes in the macrophages were observed, indicating the cellular uptake of DiI-labeled exosomes by macrophages (Fig. 1I). All analyses presented above indicated that the rat BMSC-Exos were successfully isolated and identified.

### BMSC-Exos promoted M1 macrophage transformation toward an M2-like phenotype

**Fig. 2:**
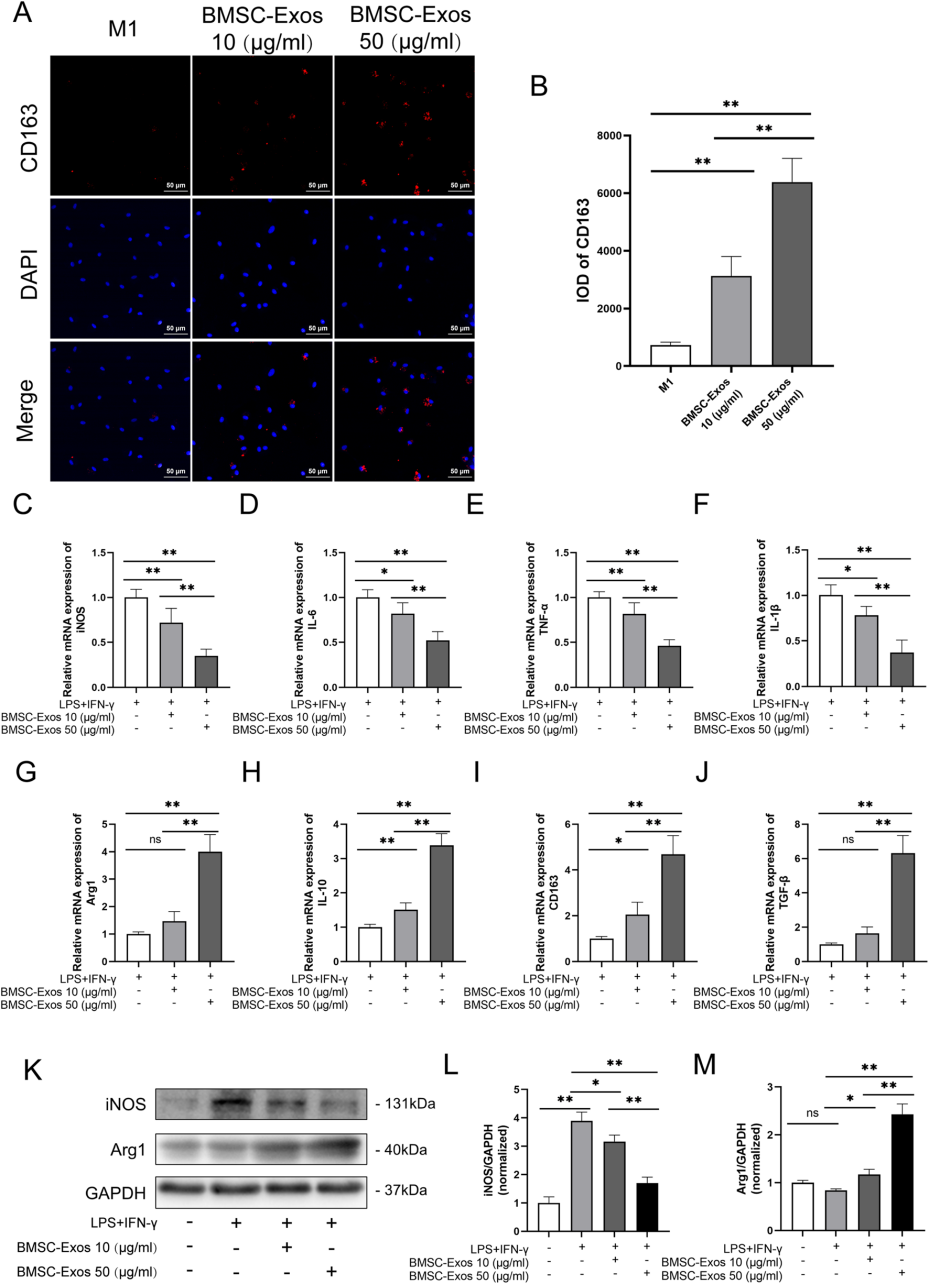
The BMSC-Exos polarized macrophages from the M1 phenotype into M2. **A**, **B** Representative fluorescence images of CD163 expression and integrated optical density (IOD) in M1 macrophages treated with 10 or 50 μg/ml BMSC-Exos. *n* = 3. **C–J** The mRNA expression of M1 markers (iNOS, IL-6, IL-1β, and TNF-α) and M2 markers (CD163, IL-10, TGF-β, and Arg1) in LPS+IFNγ-stimulated macrophages after incubation with PBS or BMSC-Exos for 48 h. *n* = 6. **K–M** Protein levels of M1 markers (iNOS) and M2 markers (Arg1) in LPS+IFNγ-stimulated macrophages after incubation with PBS or BMSC-Exos for 48 h. *n* = 3. Data are presented as mean ± SD. Statistical analysis was performed with Student’s *t* test and one-way ANOVA followed by Tukey’s multiple comparisons test. *P < 0.05, **P < 0.01.

To investigate the potential effects of BMSC-Exos on macrophages, we induced M1 macrophages using normal phenotype macrophages(M0), and then M1 macrophages were treated with different concentrations of BMSC-Exos. The results showed that in the group of M1 macrophages with PBS treatment, there was less CD163 (M2 marker) expression. After treatment with 10 μg/ml BMSC-Exos, the integrated optical density (IOD) values of CD163 increased 4.302-fold compared to the control group, which reached an 8.790-fold change with treatment with 50 μg/ml BMSC-Exos (Fig. 2A, B). Furthermore, qRT-PCR analysis of M1 and M2 gene expression also showed that after treatment with 50 μg/ml BMSC-Exos, M1 markers (iNOS, IL-6, IL-1β, and TNF-α) in macrophages were significantly reduced, whereas M2 markers, such as CD163, IL-10, TGF-β, and Arg1, were increased (Fig. 2C–J), which was consistent with Western blot analysis (Fig. 2K–M). In summary, these results demonstrated that BMSC-Exos polarized macrophages from the M1 macrophages into M2 macrophages.

### miR-23a-3p/IRF1 is a candidate pathway of BMSC-Exo-mediated macrophage inflammation

**Fig. 3:**
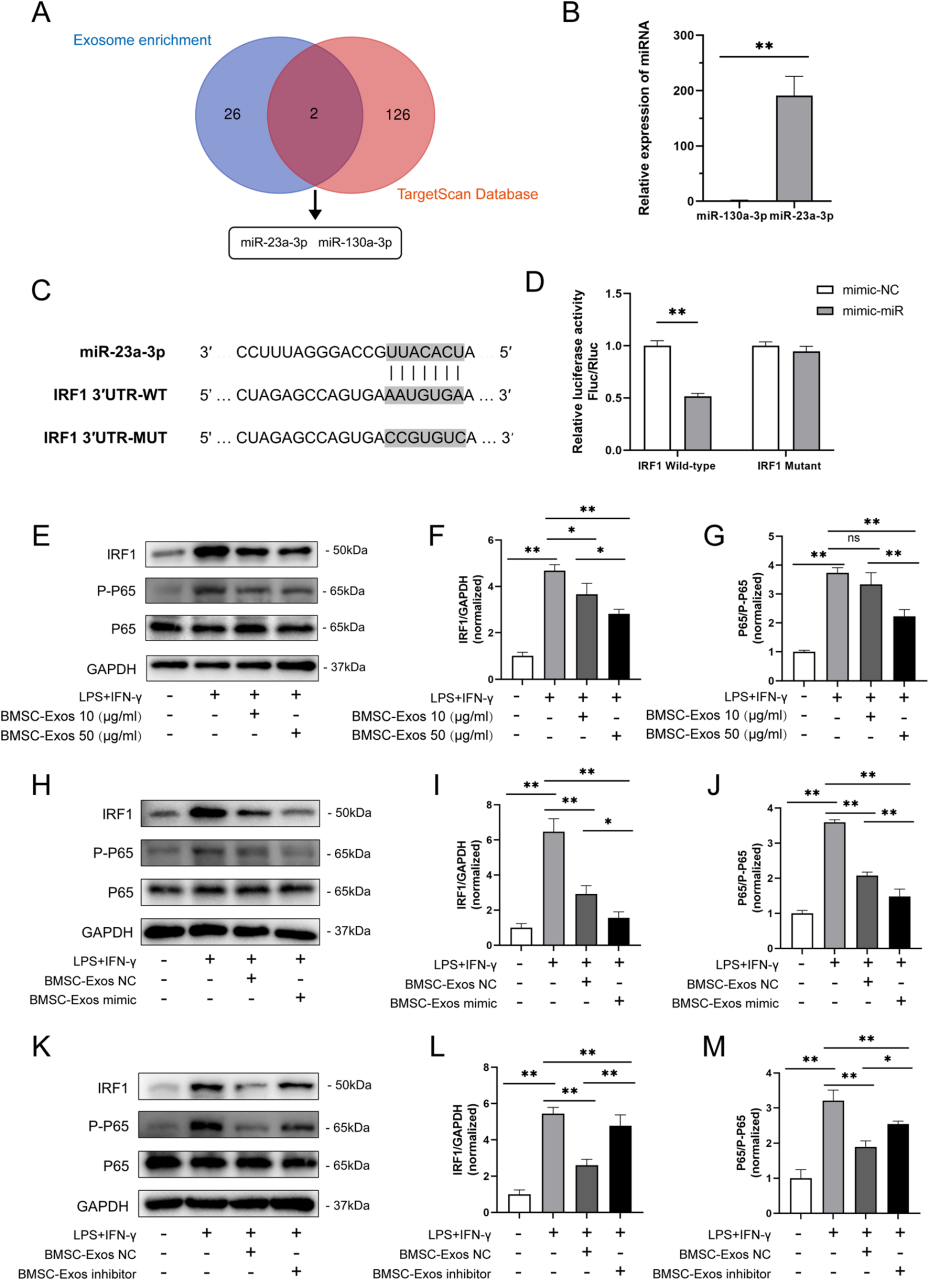
miR-23a-3p shuttling by BMSC-Exos modulates macrophage inflammation through targeting IRF1. **A** Venn diagram of the miRNA enriched in BMSCs and the miRNAs regulating IRF1. **B** The miRNA expression of miR-130-3p and miR-23a-3p in BMSC-Exos. The expression levels of the miRNAs were normalized to U6. *n* = 6. **C** The sequence of the wild-type or mutant seed region of IRF1 and the binding site of IRF1 mRNA with miR-23a-3p and the mutant 3′-UTR of IRF1. **D** 293T cells were co-transfected with luciferase reporter constructs containing wild-type/mutant 3′-UTR of IRF1 and miR-23a-3p NC/mimic for 24 h. The relative luciferase activity was normalized to Renilla luciferase luminescence. *n* = 6. **E–G** Protein levels of IRF1, P-P65, and P65 in LPS+IFN-γ-stimulated macrophages after being treated with 10 or 50 μg/ml BMSC-Exos for 48 h. *n* = 3. **H–J** Protein levels of IRF1, P-P65, and P65 in LPS+IFN-γ-stimulated macrophages after being treated with BMSC-Exos with miR-23a-3p mimic (BMSC-Exos mimic) or negative control (BMSC-Exos NC) for 48 h. *n* = 3. **K–M** Protein levels of IRF1, P-P65, and P65 in LPS+IFN-γ stimulated macrophages after being treated with BMSC-Exos with miR-23a-3p inhibitor (BMSC-Exos inhibitor) or BMSC-Exos NC for 48 h. *n* = 3. Data are presented as mean ± SD. Statistical analysis was performed with Student’s *t* test and one-way ANOVA followed by Tukey’s multiple comparisons test. ^*^P < 0.05, ^**^P < 0.01.

miRNAs are highly conserved small non-coding RNAs composed of 19–24 nucleotides that can regulate gene expression at the posttranscriptional level and induce target gene silencing [28]. Studies have reported that exosomes have an essential role in cellular communication through the exchange of miRNAs or proteins between cells [29]. Therefore, we next investigated whether BMSC-Exos contained any pivotal miRNAs that contributed to their anti-inflammatory effect. IRF1 is the first reported member of the IRF family [30–32], which has been reported to be a crucial regulator in inflammation and M1 macrophage polarization [33–36]. Using the TargetScan database, we found 128 miRNAs that can regulate IRF1. Then, these miRNAs were aligned to the published miRNA sequencing data on BMSC-Exos by Ferguson. et al. [37] After analysis, we found that miR-23a-3p and miR-130a-3p stood out as candidates enriched in BMSC-Exos probably responsible for regulating IRF1 (Fig. 3A). qRT-PCR further confirmed that miR-23a-3p was more abundant than miR-130a-3p in BMSC-Exos (Fig. 3B). Next, a luciferase reporter gene assay was used to verify the regulatory relationship of miR-23a-3p on IRF1. The results showed that the miR-23a-3p mimic obviously reduced the expression of wild-type IRF1 but did not affect mutant IRF1 (Fig. 3D) (information on the wild-type and mutant IRF1 is shown in Fig. 3C). Furthermore, Western blot analysis revealed that the levels of P-P65 and IRF1 in the NF-κB pathway were upregulated in M1 macrophages and were significantly downregulated after treatment with 10 or 50 µg/ml BMSC-Exos or miR-23a-3p mimic BMSC-Exos (BMSC-Exos mimic) (Fig. 3E–J). However, the effect of BMSC-Exos decreased after transfection with the miR-23a-3p inhibitor (Fig. 3K–M). These results demonstrated that miR-23a-3p might be the key regulatory cargo contained in BMSC-Exos accounting for the regulation of IRF1.

### miR-23a-3p in BMSC-Exos mediated macrophage polarization

**Fig. 4:**
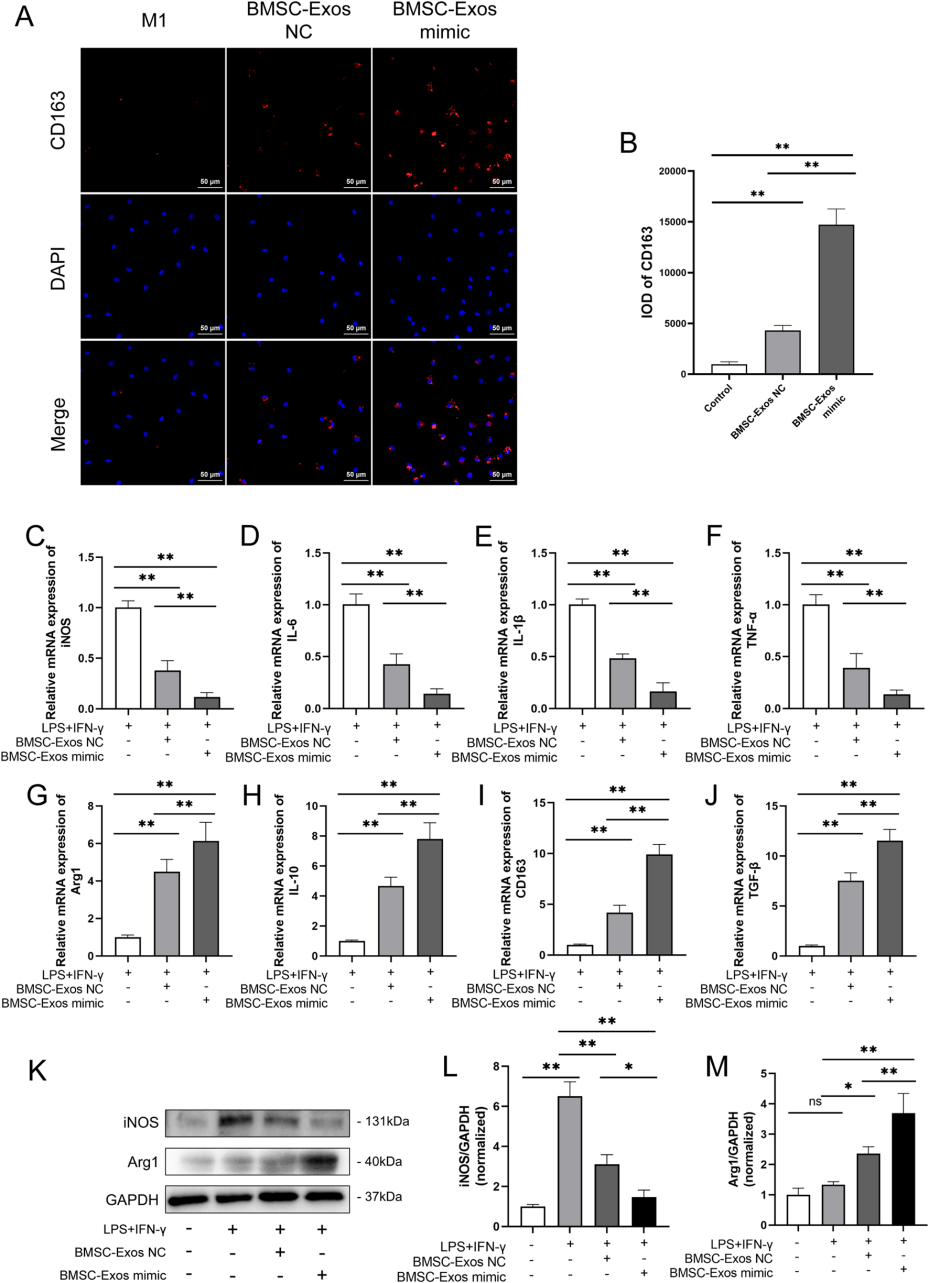
BMSC-Exos mediated macrophage polarization via miR-23a-3p. **A**, **B** Representative fluorescence images of CD163 expression and integrated optical density (IOD) in M1 macrophages treated with BMSC-Exos mimic or BMSC-Exos NC for 48 h. *n* = 3. **C–J** The mRNA expression of M1 markers (iNOS, IL-6, IL-1β, and TNF-α) and M2 markers (CD163, IL-10, TGF-β, and Arg1) in LPS+IFNγ stimulated macrophages after incubation with BMSC-Exos NC or BMSC-Exos mimic for 48 h. *n* = 6. **K–M** Protein levels of M1 markers (iNOS) and M2 markers (Arg1) in LPS+IFNγ-stimulated macrophages after incubation with BMSC-Exos NC or BMSC-Exos mimic for 48 h. *n* = 3. Data are presented as mean ± SD. Statistical analysis was performed with Student’s *t* test and one-way ANOVA followed by Tukey’s multiple comparisons test. *P < 0.05, **P < 0.01.

To understand whether miR-23a-3p from BMSC-Exos could regulate macrophage polarization, we detected variation in the marker of M2 macrophages after treatment with BMSC-Exos mimic. Compared with M1 macrophage treatment, BMSC-Exos NC treatment promoted CD163 expression in M1 macrophages, while BMSC-Exos mimic resulted in further enhancement of CD163 expression (Fig. 4A, B). In addition, qRT-PCR analysis of M1 and M2 gene expression also showed that after treatment with BMSC-Exos mimic, M1 markers (iNOS, IL-6, IL-1β, and TNF-α) in M1 macrophages were significantly reduced, whereas M2 markers, such as CD163, IL-10, TGF-β, and Arg1, were increased compared to PBS or BMSC-Exos NC (Fig. 4C–J). Western blotting analysis confirmed that BMSC-Exos NC or BMSC-Exos mimic promoted M2 marker expression but reduced M1 marker expression, and BMSC-Exos mimic had a more obvious effect than normal BMSC-Exos (Fig. 4K–M). These results suggested that miR-23a-3p in BMSC-Exos mediated macrophage polarization.

### ACLR rat model establishment and biomechanical analysis

**Fig. 5:**
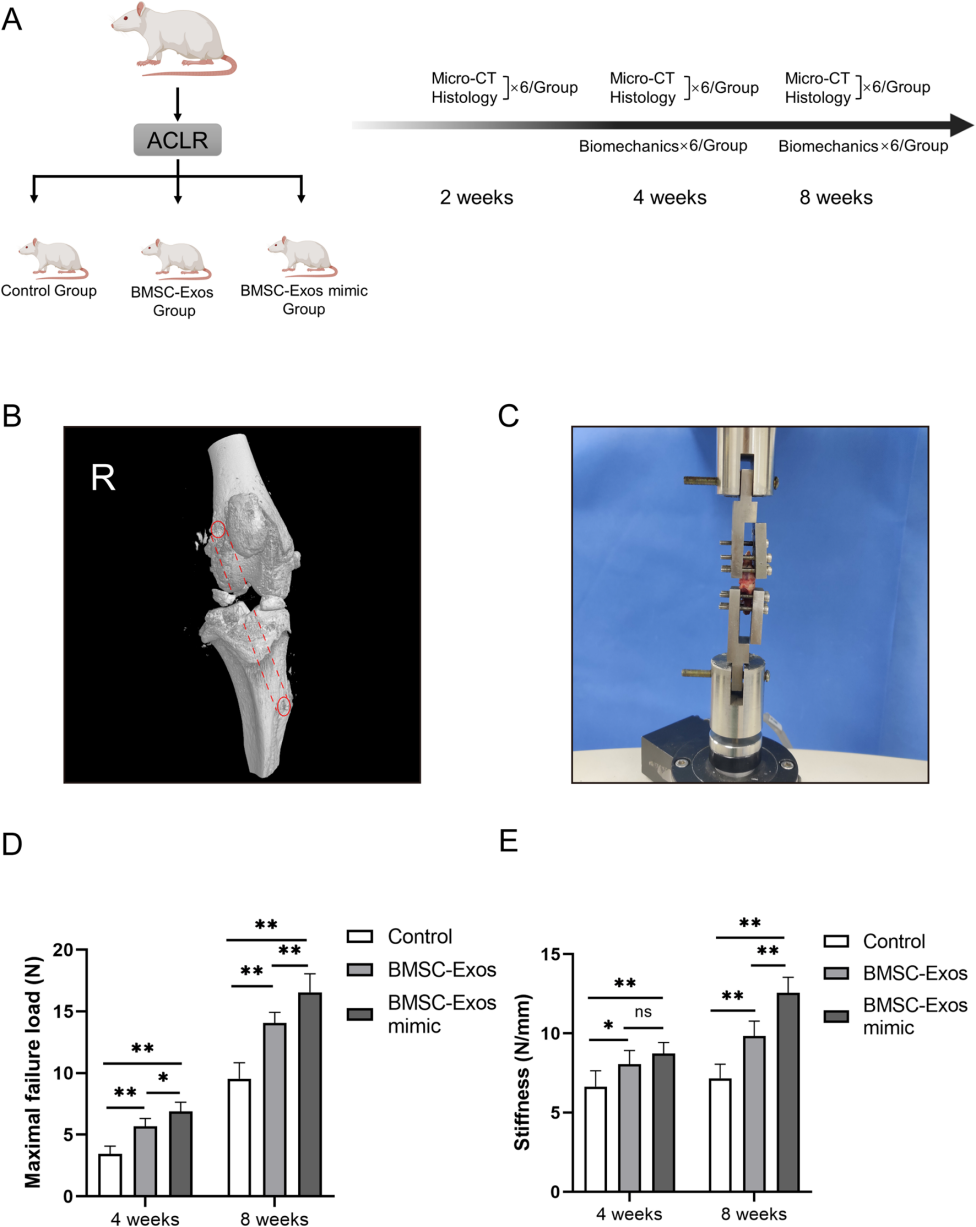
Biomechanical testing of the ACLR tissue after the treatment of BMSC-Exos. **A** Flowchart of the group allocation and analysis items at each time point. **B** The scheme of the ACLR operation. **C** Biomechanical test of the reconstruction ACLR tissue using a mechanical tester. **D** The maximal failure load and **E** stiffness of the femur-graft-tibia complex after ACLR at 4 and 8 weeks postoperatively among the control group, the BMSC-Exos group and the BMSC-Exos mimic group. Data are presented as mean ± SD. Statistical analysis was performed with one-way ANOVA followed by Tukey’s multiple comparisons test. *n* = 6. *P < 0.05, **P < 0.01.

Next, to verify the potential mechanism of BMSC-Exos on tendon-bone healing in vivo, we established an ACLR rat model. Fig. 5A shows the animal experimental design scheme, and Fig. 5B shows the three-dimensional (3D) structure of the bone tunnel schematic diagram. Refer to the description in the Methods section for operation details. First, we detected the healing of tendons to bone after ACL reconstruction through biomechanical tests at 4 and 8 weeks after the operation using a mechanical tester (Fig. 5C). After separating the redundant tissues of the knee joint and leaving only the reconstructed ACL, the tissues were fixed firmly on a mechanical tester. Based on this system, the maximum failure force and stiffness of the tissue can be detected, and the healing degree of the tendon-bone interface can be inferred. The results showed that at both 4 weeks and 8 weeks, the BMSC-Exos and the BMSC-Exos mimic significantly enhanced the maximal failure load compared with the control group, and the BMSC-Exos mimic group performed more effectively compared with the BMSC-Exos group (Fig. 5D). Simultaneously, we found a similar promotion effect of the BMSC-Exos and the BMSC-Exos mimic on the stiffness of the reconstructed tissues (Fig. 5E). There was no remarkable difference between the BMSC-Exos and the BMSC-Exos mimic at 4 weeks. These results indicated that miR-23a-3p in BMSC-Exos played an important role in the mechanical properties of ACLR tissue.

### Micro-CT examinations of the area of bone tunnels at the joint surface

**Fig. 6.**
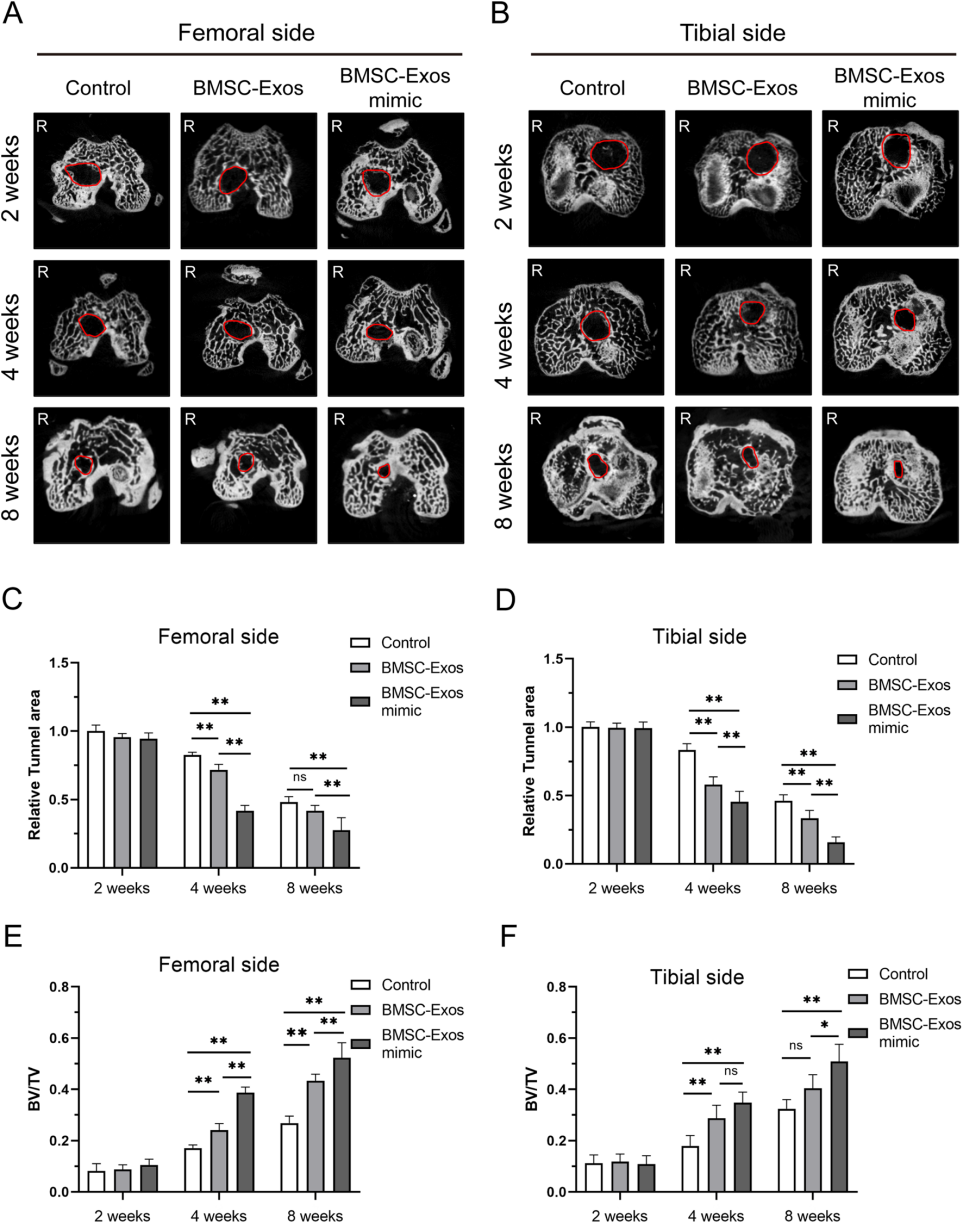
Micro-CT imaging analysis of the different groups at 2, 4, and 8 weeks postoperatively. The cross-sectional micro-CT images of the bone tunnel at the femoral side (**A**) and tibial side (**B**). Relative tunnel area at the femoral (**C**) and tibial (**D**) sides and the bone volume/total volume ratio (BV/TV) at the femoral (**E**) and tibial (**F**) sides for ACLR at 2, 4, and 8 weeks postoperatively among the control group, the BMSC-Exos group and the BMSC-Exos mimic group. Data are presented as mean ± SD. Statistical analysis was performed with one-way ANOVA followed by Tukey’s multiple comparisons test. *n* = 6. *P < 0.05, **P < 0.01.

The area of both the femoral and tibial bone tunnels at the joint surface postoperatively was analyzed by micro-CT (Fig. 6A, B). The differences in tunnel area reduction at both the femoral and tibial sides were normalized to that of the control group at 2 weeks and were further compared.

For the tunnel area, there was no significant difference among the groups on either the femoral or tibial side at 2 weeks postoperatively (Fig. 6C, D). At 4 weeks postoperatively, the tunnel area both at femoral and tibial side was significantly less in the BMSC-Exos group (71.61%, 57.91%, respectively) or the BMSC-Exos mimic group (41.62%, 45.38%, respectively) than in the control group (82.51%, 83.22%, respectively), and the BMSC-Exos mimic group has the significant less tunnel area compared with the BMSC-Exos group (Fig. 6C, D). At 8 weeks postoperatively, the tunnel area among the control group (48.04%, 46.18%, respectively), the BMSC-Exos group (41.62%, 33.39%, respectively) and the BMSC-Exos mimic group (27.53%, 15.79%, respectively) was further reduced, and the tunnel area at the tibial side was significantly less in the BMSC-Exos group or the BMSC-Exos mimic group than in the control group (*P* <0.001, *P* <0.001, respectively), and the BMSC-Exos mimic group has the significant less tunnel area compared with the BMSC-Exos group (Fig. 6D). Similarly, the tunnel area in the BMSC-Exos mimic group on the femoral side was significantly less than the tunnel area in the control group or in the BMSC-Exos group (*P* = 0.001, *P* = 0.004, respectively) (Fig. 6C). However, there was no significant difference between the BMSC-Exos group and the control group.

Mineralized tissue formation in the intra tunnel portion of the scaffold (BV/TV) was also measured. Generally, the BV/TV increased from 2 to 8 weeks in all groups (Fig. 6E, F). There was no significant difference among the groups on either the femoral or tibial side at 2 weeks postoperatively (Fig. 6E, F). At 4 weeks postoperatively, compared with the control group, the BV/TV value of the bone tunnel in the BMSC-Exos group and the BMSC-Exos mimic group was significantly increased on both the femoral and tibial sides. However, compared with the BMSC-Exos group, the BV/TV value of the bone tunnel in the BMSC-Exos mimic group was significantly enhanced on the femoral side, but there were no obvious changes on the tibial side (Fig. 6E, F). At 8 weeks postoperatively, the BV/TV value of the bone tunnel in the BMSC-Exos mimic group on both the femoral and tibial sides was significantly higher than the BV/TV value in the control group or in the BMSC-Exos group (Fig. 6E, F). However, compared with the control group, the BV/TV value of the bone tunnel in the BMSC-Exos group was significantly enhanced on the femoral side, but there were no obvious changes on the tibial side (Fig. 6E, F).

In summary, more mineralized tissue was formed in the bone tunnels in the BMSC-Exos group and the BMSC-Exos mimic group on both the femoral and tibial sides, and the BMSC-Exos mimic showed more effectiveness than the BMSC-Exos.

### Histological analysis on ACLR postoperative

**Fig. 7:**
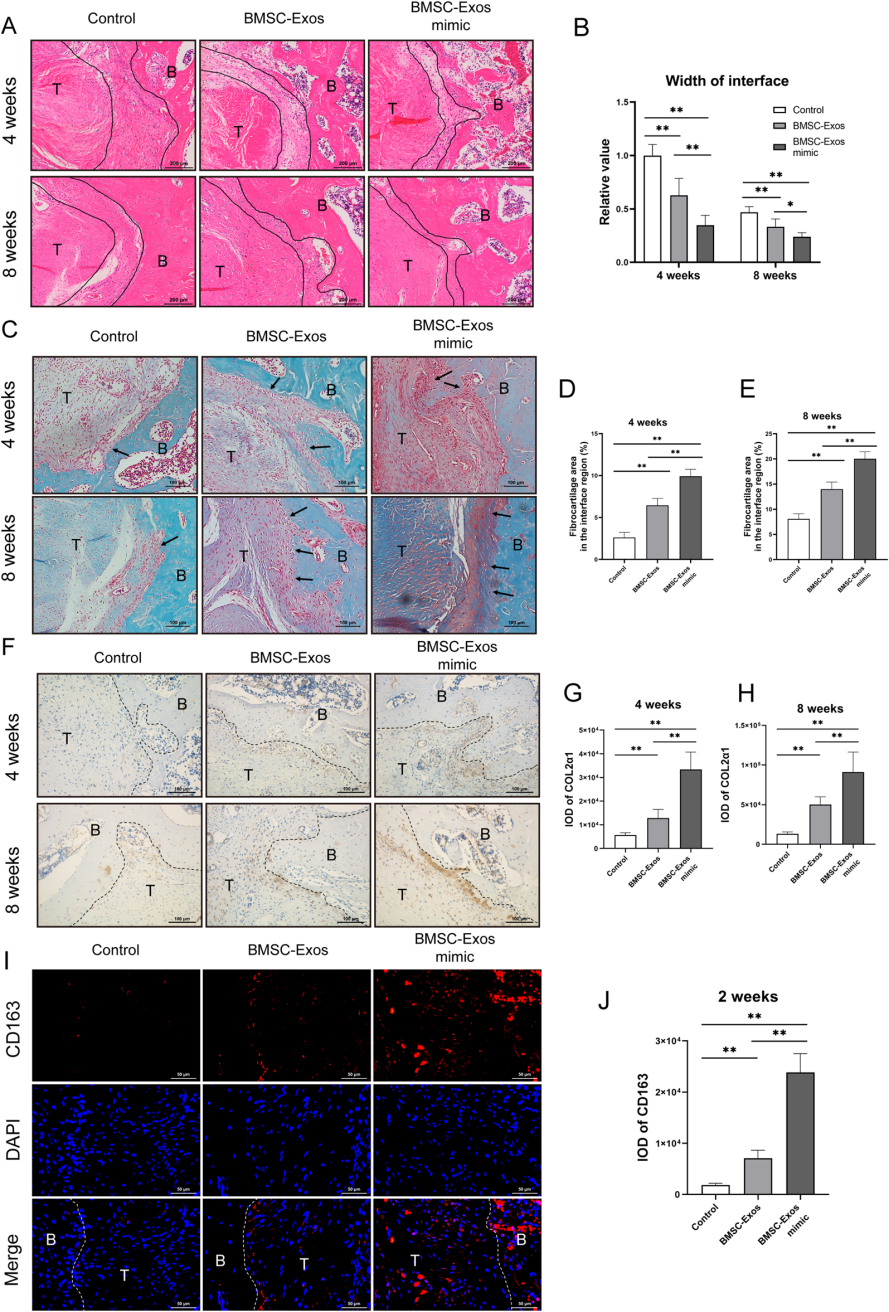
Histological analysis on ACLR postoperative. **A** Representative image of the tendon-bone interface by hematoxylin and eosin staining at 4 and 8 weeks postoperatively among the control group, the BMSC-Exos group and the BMSC-Exos mimic group, with quantification of the relative width of the interface. Scale bar: 200 μm. **B** The interface is outlined by the black line. **C–E** Safranin-O/Fast Green staining at the tendon-bone interface and quantification of fibrocartilage in the interface region after ACLR at 4 and 8 weeks postoperatively among the control group, the BMSC-Exos group and the BMSC-Exos mimic group. Scale bar: 100 μm. **F**, **H** Immunohistochemical staining of COL2α1 at the tendon-bone interface and quantification after ACLR at 4 and 8 weeks postoperatively among the control group, the BMSC-Exos group and the BMSC-Exos mimic group. Scale bar: 100 μm. **I**, **J** Immunofluorescence staining of CD163 at the tendon-bone interface and quantification after ACLR at 2 weeks postoperatively among the control group, the BMSC-Exos group and the BMSC-Exos mimic group. Scale bar: 50 μm. B, bone; T, tendon graft. Data are presented as mean ± SD. Statistical analysis was performed with one-way ANOVA followed by Tukey’s multiple comparisons test. *n* = 6. ^*^P < 0.05, ^**^P < 0.01.

Finally, we examined the histological changes in ACLR tissues postoperatively. The H&E staining results showed that a distinct tendon-bone interface was noticed in all 3 groups at 4 weeks with disordered fibrovascular tissue (Fig. 7A). Furthermore, compared with the control group, the relative width of the interface between the host bone and graft at 4 and 8 weeks was also significantly reduced in the BMSC-Exos group and the BMSC-Exos mimic group, and the relative width of the interface was obviously shorter than the relative width of the former group (Fig. 7B). Moreover, there was a decreasing trend in the relative width of the interface at 4 weeks between the BMSC-Exos mimic group and the BMSC-Exos group, which was remarkably decreased at 8 weeks in the BMSC-Exos mimic group (Fig. 7B). An increased formation of chondrocytes and fibrocartilage at the tendon-bone interface indicates better tendon-bone healing [38, 39]. Our results indicated that BMSC-Exos could increase chondrocytes and fibrocartilaginous tissues at the tendon-bone interface, while the BMSC-Exos mimic group showed more formation of chondrocytes and fibrocartilaginous tissues at 4 and 8 weeks after operation (Fig. 7C–E). To quantify the level of collagen fibril remodeling, the COL2α1 protein was detected by IHC. The results showed that compared with the control group, the positive staining of COL2α1 was significantly increased at 4 and 8 weeks in both the BMSC-Exos mimic group and the BMSC-Exos group, and the staining intensity of the former group was obviously stronger than the staining intensity of the latter group (Fig. 7F–H). We hypothesized that early postoperative inflammation inhibition may promote early tendon-bone healing in the BMSC-Exos mimic group. The IF staining of a general macrophage marker (CD68) (Additional file 1: Fig. S1) showed that BMSC-Exos and BMSC-Exos mimic treatment did not show significant differences in total macrophage numbers in the ACLR rat model. However, compared with the control group or the BMSC-Exos group, the M2 macrophages marker CD163 was significantly enhanced in the BMSC-Exos mimic group, indicating that the polarization of M2 macrophages occurred at the tendon-bone interface in the BMSC-Exos mimic group in the early postoperative stage, which inhibited the inflammatory response (Fig. 7I, J). The above results suggested that the BMSC-Exos mimic can inhibit the early postoperative inflammatory reaction and promote tendon-bone healing after ACLR.

## Discussion

In this study, we first found that BMSC-Exos could regulate the polarization of macrophages to promote tendon-bone healing in rats after ACLR. The specific mechanism was that miR-23a-3p in BMSCs-Exos could inhibit IRF1 expression and the NF-κB signaling pathway in macrophages, promote the polarization of M1 macrophages to M2 macrophages and inhibit local inflammation at the tendon-bone interface. This study provided new methods and new directions for acellular therapy for tendon-bone healing after ACLR.

The ACL is an important structure to maintain the stability of the knee joint. After ACL tears, patients have a significantly higher risk of secondary meniscal tears, knee joint instability, traumatic arthritis, cartilage damage, etc. [40]. Although the development of arthroscopic techniques has created favorable conditions for ACLR in recent years, as the main repair method, ACLR with the hamstring tendon still has problems such as a long period of tendon-bone healing, poor tendon-bone healing and insufficient local biomechanical strength [41–44]. Therefore, how to accelerate tendon-bone healing after ACLR is a common concern of doctors and researchers around the world.

Fortunately, people found that stem cells have enormous potential for tissue repair. BMSCs are adult stem cells with self-replication and multi-differentiation potential derived from bone marrow that can repair damaged tissue and are regarded as the most promising candidates for regenerative medicine and tissue engineering [45, 46]. The therapeutic functions of BMSCs have been extensively studied in tissue engineering repair areas, such as cartilage, heart, lung, and kidney repair [47, 48]. Although there have been some exciting advances in cellular therapy of BMSCs, the mechanism has not yet been fully understood. Recent studies have indicated that exosomes containing various biologically active substances, such as miRNAs and proteins, may be the key delivery for BMSCs to exert their therapeutic effects [12]. Exosomes are double-layer membranous bodies; therefore, the biologically active substances contained in exosomes can play a role through the endocytosis of exosomes [29]. Studies have shown that, compared to BMSCs themselves, purified BMSCs-Exos have more unique advantages in damaged tissue repair: a) better stability: the exosomes are coated with phospholipid membranes on their outer surfaces, which can ensure that the various bioactive components contained in exosomes are not easily degraded by enzymes and can be preserved for a long time without changing their bioactivity; b) fast transportation and strong permeability. c) good biocompatibility, which can avoid the immune response and tumorigenesis of BMSCs [12, 49]. Therefore, acellular therapies based on BMSC-Exos may replace BMSCs as a more promising treatment. So, can BMSC-Exos be used to promote tendon-bone healing after ACLR?

It is known that early local inflammation after ACLR profoundly affects the bone healing process of ACLR, and macrophages play an important role in this process [7, 50]. Macrophages are natural immune cells that originate from monocytes in the blood. When the body is stimulated by trauma, monocytes migrate to the trauma site and transform into macrophages, which regulate the development of tissue inflammation [51]. Macrophages have been found to have good plasticity and versatility. Under different microenvironments in vivo, they can exhibit different phenotypes and show other functions. This process is called macrophage polarization [52]. In the early stage after ACLR operation, macrophages are polarized into classically activated macrophages, namely M1 macrophages, and many proinflammatory factors, such as tumor necrosis factor α (TNF-α), IL-12 and iNOS, are highly expressed to enhance the local inflammatory response [7]. This inflammatory microenvironment is conducive to recruiting more cells from other parts to reach the damaged site to play an effect and prepare for subsequent tissue repair. Subsequently, macrophages were polarized to replace activated macrophages, namely M2 macrophages, and many anti-inflammatory factors, such as IL-10 and transforming growth factor β (TGF-β), were highly expressed, thereby reducing the local inflammatory response and promoting local tissue regeneration and repair [53]. After ACLR, tendon-bone healing experienced graft necrosis and vascularization, fibrous tissue connection replacement, new bone formation, bone into tendon, remodeling and other stages. In the early stage after reconstruction, the tendon-bone interface showed an acute inflammatory response. Numerous macrophages infiltrated, and local hematomas and fibroblasts formed granulomas with a large amount of collagen deposition, resulting in the formation of a large number of local scar tissues [54]. Compared with the original structure, scar tissue affects the regeneration of new bone and the growth of bone into the tendon, and its function of transmitting load and dispersing stress is significantly weakened, which reduces the quality of tendon-bone healing [55]. Therefore, we hypothesized that inhibition of the early inflammatory response mediated by macrophages after ACLR may contribute to tendon-bone healing.

Studies have shown that miRNAs in exosomes derived from BMSCs can modify the polarization of M1 macrophages to M2 macrophages to attenuate tissue injury, such as myocardial ischemia–reperfusion injury [16] and acute lung injury [56]. Recently, some researchers have found that miR-23a-3p has a high content of BMSC-Exos [37, 57]. Consistently, our results also show that BMSC-Exos highly expressed miR-23a-3p, and we found that miR-23a-3p can target the 3’UTR of IRF1 by bioinformatics analysis. IRF1 has been reported to be a crucial regulator in inflammations and M1 macrophage polarization. For example, IRF1 knockdown dramatically regulates M2 macrophage polarization and alleviates inflammation [33, 35, 36], while IRF1 activation can effectively promote the expression of iNOS (a marker of M1 macrophages) in macrophages [34]. Therefore, we hypothesized that miR-23a-3p in BMSC-Exos might regulate the polarization of M2 macrophages by targeting and inhibiting the expression of IRF1. In this study, *in vitro* experiments confirmed that BMSC-Exos could inhibit IRF1 expression and the NF-κB signaling pathway and then promote M2 polarization, and the overexpression of miR-23a-3p could further enhance the regulatory effect of BMSC-Exos on M2 polarization. These results indicated that miR-23a-3p in BMSC-Exos could inhibit IRF1 expression in macrophages, thereby inhibiting the inflammatory response and promoting the polarization of M2 macrophages.

Signs of tendon-bone healing are bone fusion, improved mechanical level and reduction of the bone tunnel [25, 58, 59]. In the process of the tendon-bone repair interface, the tendon-bone interface usually includes four kinds of tissues: fibrous connective tissue, non-calcified fibrous cartilage, calcified fibrous cartilage and bone tissue. Non-calcified and calcified fibrocartilage tissues are composed mainly of type II collagen [60], while early cartilage regeneration and fibrocartilage formation are the key factors in the process of tendon-bone healing[61, 62]. In this study, we administered intra-articular injection of exosomes for the treatment after ACLR, which has been proven to be an effective method for exosome delivery [63, 64]. The results demonstrated that the maximal failure load of each group increased with time and the maximal failure load of the BMSC-Exos group was significantly enhanced compared with the maximal failure load of the control group, while miR-23a-3p overexpression in BMSC-Exos further enhanced this effect. Micro-CT was used to measure the area of the bone tunnel, and BV/TV was used to measure bone formation around the graft. The results showed that compared with the control group, the BMSC-Exos group showed a decreased relative area of the bone tunnel and increased BV/TV. At 8 weeks, the BMSC-Exos mimic group showed a smaller relative area of the bone tunnel and higher BV/TV than BMSC-Exos group. In addition, at 8 weeks, H&E staining results showed that compared with the control group and the BMSC-Exos group, the relative width of the interface between the bone and graft was also significantly reduced in the BMSC-Exos mimic group. IHC staining showed that COL2α1 expression in the BMSC-Exos mimic group was significantly higher than the COL2α1 expression in the other two groups at 4 and 8 weeks after ACLR, indicating that early fibrocartilage regeneration was enhanced. In addition, we found that M2 macrophages marker (CD163) but not the macrophages marker (CD68) was significantly increased in the BMSC-Exos group and BMSC-Exos mimic group, indicating that the polarization of M2 macrophages occurred at the tendon-bone interface in the BMSC-Exos mimic group in the early postoperative stage, which inhibited the inflammatory response, while BMSC-Exos or BMSC-Exos mimic did not influence the macrophage proliferation. In summary, we concluded that the early administration of BMSC-Exos with miR-23a-3p overexpression promoted the tendon-bone healing of ACLR, indicating that early BMSC-Exos treatment could inhibit the inflammatory response of the tendon-bone interface and promote the regeneration of fibrocartilage to accelerate tendon-bone healing after ACLR, and the therapeutic effect could be enhanced by the overexpression of miR-23a-3p.

There were still some limitations in this study. The postoperative long-term effect of BMSC-Exos treatment was not evaluated. In addition, it is worthy for modifying exosomes to improve their targeting effect on macrophages to achieve the precise regulation of M2 macrophages. Moreover, in addition to non-coding RNA, other bioactive substances in exosomes, such as specific components of lipids and proteins, also play an essential role in cellular communication, which are predicted to be new disease biomarkers [65, 66]. Therefore, it is necessary to further explore the wider role of exosomes in tendon-bone healing after ACLR surgery.

## Conclusions

Generally, we confirmed that miR-23a-3p in BMSC-Exos promoted the polarization of M2 macrophages and reduced the inflammatory response of the tendon-bone interface by targeting the inhibition of the IRF1 and NF-κB pathway in macrophages and promoted tendon-bone healing after ACLR. This study provides theoretical and experimental evidence for stem cell therapy for early tendon-bone healing after ACLR.

## Supplementary Information

**Additional file 1: Fig. S1.** Histological analysis of total macrophages ACLR postoperative.

## Data Availability

The datasets used and/or analyzed during the current study are available from the corresponding author on reasonable request.

## Abbreviations

3D: Three-dimensional
ACL: Anterior cruciate ligament
ACLR: Anterior cruciate ligament reconstruction
Arg1: Arginase 1
BMDMs: Bone marrow derived macrophages
BMSC: Bone marrow stromal cell
BMSC-Exos mimic: BMSC-Exos with miR-23a-3p overexpression
BMSC-Exos NC: BMSC-Exos transfected with negative control
BMSC-Exos inhibitor: BMSC-Exos with miR-23a-3p inhibitor
BMSC-Exos: Bone marrow stromal cell-derived exosomes
BV/TV: Bone volume/total volume ratio
COL2α1: Collagen type II alpha 1
DAPI: 4′,6-Diamidino-2-phenylindole
DiI: 1,1′-Dioctadecyl-3,3,3′,3′-tetramethylindocarbocyanine perchlorate
EDTA: Ethylenediaminetetraacetic acid
FBS: Fetal bovine serum
GAPDH: Glyceraldehyde-3-phosphate dehydrogenase
HE: Hematoxylin eosin staining
IF: Immunofluorescence
IFN-γ: Interferon-gamma
IHC: Immunohistochemistry
IL-10: Interleukin 10
IL-1β: Interleukin 1β
IL-6: Interleukin 6
iNOS: Inducible nitric oxide synthase
IOD: Integrated optical density
IRF1: Interferon regulatory factor 1
LPS: Lipopolysaccharides
M-CSF: Macrophage colony stimulating factor
NTA: Nanoparticle tracking analysis
qPCR: Quantitative polymerase chain reaction
TEM: Transmission electron microscopy
TGF-β: Transforming growth factor β
TNF-α: Tumor necrosis factor α
TSG101: Tumor susceptibility gene 101
