# Supplementary material for: BMSC-derived exosomes promote tendon-bone healing after anterior cruciate ligament reconstruction by regulating M1/M2 macrophage polarization in rats
Source: Stem Cell Res Ther. 2022 Jul 15;13:295. doi: 10.1186/s13287-022-02975-0 (PMC9284827; doi:10.1186/s13287-022-02975-0)
Supplement: Supplementary file 1 — Additional file 1: Fig. S1. Histological analysis of total macrophages ACLR postoperative. [file 13287_2022_2975_MOESM1_ESM.docx]

**BMSC-derived exosomes promote tendon-bone healing after anterior cruciate ligament reconstruction by regulating M1 macrophage polarization in rats**

Zhenyu Li^a#^, Qingxian Li^a#^, Kai Tong^a^, Jiayong Zhu^a^, Hui Wang^b,c^, Biao Chen^a*^, Liaobin Chen^a,c*^

^a^ Division of Joint Surgery and Sports Medicine, Department of Orthopedic Surgery, Zhongnan Hospital of Wuhan University

^b^ Department of Pharmacology, Basic Medical School of Wuhan University, Wuhan, 430071, China.

^c^ Hubei Provincial Key Laboratory of Developmentally Originated Disease, Wuhan, 430071, China

^#^ Zhenyu Li and Qingxian Li contributed equally to this work.

**Corresponding authors:**

^*^ Liaobin Chen, M.D.&Ph.D., Division of Joint Surgery and Sports Medicine, Department of Orthopedic Surgery, Zhongnan Hospital of Wuhan University. E-mail: lbchen@whu.edu.cn.;

^*^ Biao Chen, M.D.&Ph.D., Division of Joint Surgery and Sports Medicine, Department of Orthopedic Surgery, Zhongnan Hospital of Wuhan University. chenbiao20030701@163.com

**
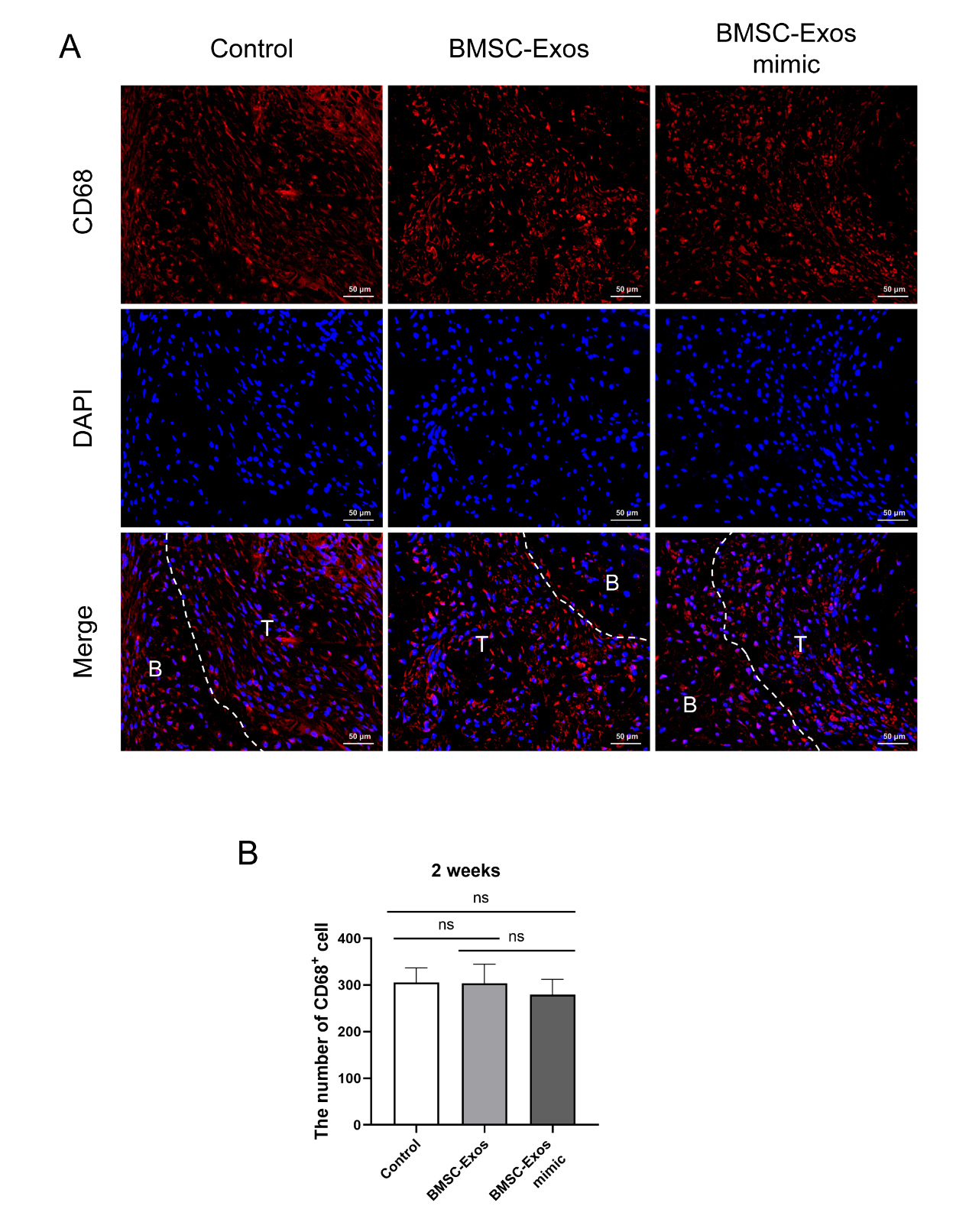
**

**Fig. S1.** Histological analysis of total macrophages ACLR postoperative. **(A)** Immunofluorescence staining of CD68 at the tendon-bone interface after ACLR at 2 weeks postoperatively among the control group, the BMSC-Exos group and the BMSC-Exos mimic group. Scale bar: 50 μm. **(B)** The number of CD68^+^ cell per visual field. B, bone; T, tendon graft. Data are presented as mean ± SD. Statistical analysis was performed with one-way ANOVA followed by Tukey’s multiple comparisons test. n = 6. not signifcant (ns)


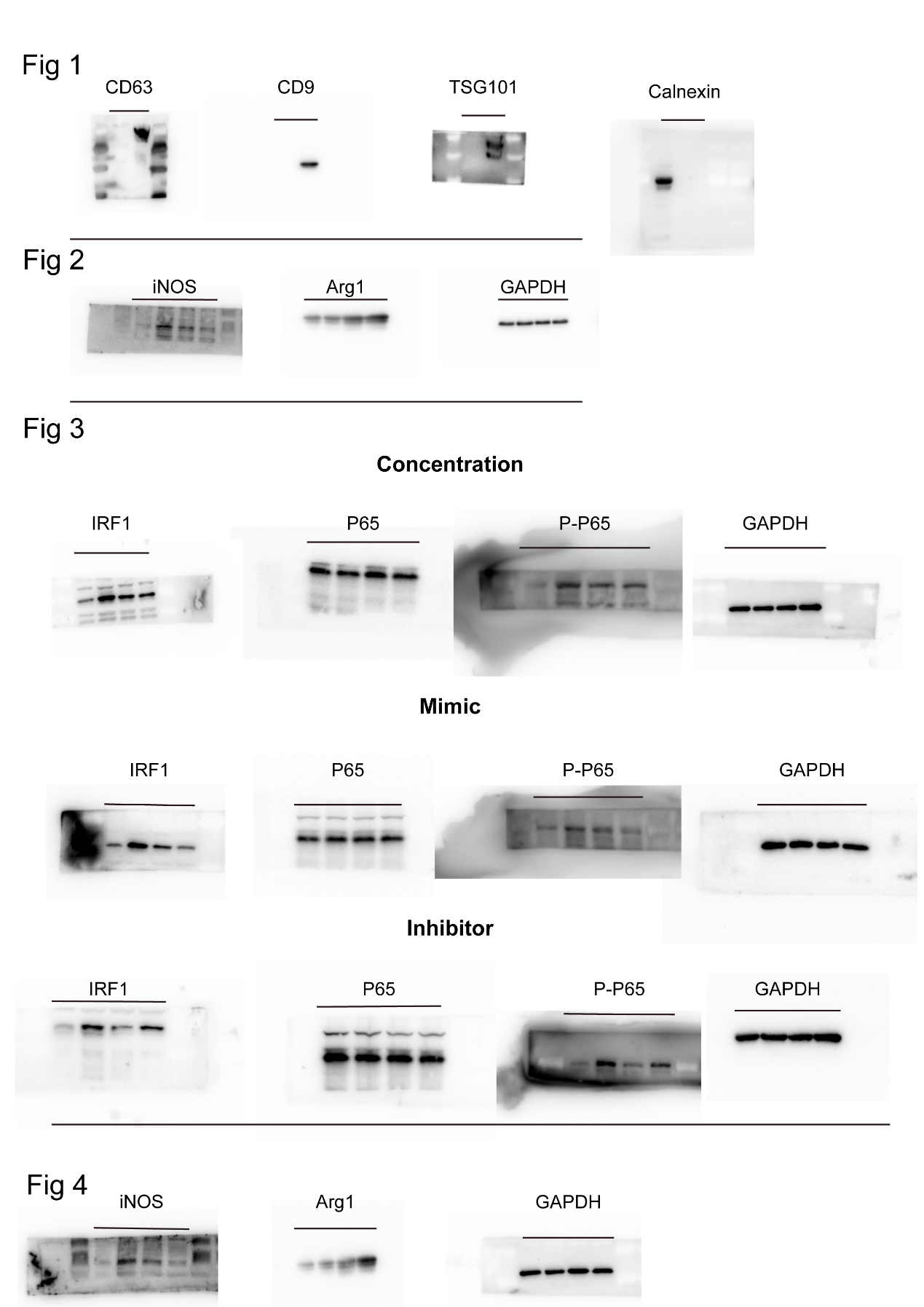
**Supplementary figure. The full-length gels and blots displayed in this study.** All the original images of the blots in the manuscript are displayed.
